# Draft Genome Sequence of the Commercial Biocontrol Strain *Pantoea agglomerans* P10c

**DOI:** 10.1128/genomeA.01448-15

**Published:** 2015-12-10

**Authors:** Theo H. M. Smits, Fabio Rezzonico, Jochen Blom, Alexander Goesmann, Azzurra Abelli, Roberto Kron Morelli, Joël L. Vanneste, Brion Duffy

**Affiliations:** a Environmental Genomics and Systems Biology Research Group, Institute for Natural Resource Sciences, Zürich University of Applied Sciences ZHAW, Wädenswil, Switzerland; b Bioinformatics and Systems Biology, Justus-Liebig-Universität, Giessen, Germany; c Agrifutur, Alfianello (Brescia), Italy; d The New Zealand Institute for Plant & Food Research Limited, Ruakura, Hamilton, New Zealand

## Abstract

We report here the draft genome sequence of the biocontrol strain *Pantoea agglomerans* P10c, composed of a draft chromosome and two plasmids: the 559-kb large *Pantoea* plasmid 1 (pPag3) and a 182-kb plasmid (pPag1). A genomic island containing pantocin A biosynthesis genes was identified.

## GENOME ANNOUNCEMENT

*Pantoea agglomerans* is a ubiquitous bacterial species often found in association with plants. Several strains of *P. agglomerans* have been found to control fungal or bacterial postharvest diseases (1, 2) and to reduce disease incidence in the orchards and fields, for example, basal kernel blight of barley caused by *Pseudomonas syringae* pv. syringae (3) or fire blight caused by *Erwinia amylovora* (2, 4, 5). *P. agglomerans* is one of the most common bacteria isolated from fire blight-susceptible plant species (4). The mechanisms by which *P. agglomerans* suppresses plant pathogens include nutritional competition and preemptive exclusion in combination with a variety of antibacterial organic acids and peptide antibiotics (5–9).

*P. agglomerans* P10c was isolated from pear blossoms in New Zealand. Selected for its exceptional ability to rapidly colonize apple and pear flowers and to suppress fire blight disease (10), it has been developed as a commercial biocontrol agent (Blossom Bless; Agrinova NZ Limited). Whole-genome sequencing (Illumina MiSeq 2 × 300-bp shotgun sequencing) yielded 2,693,476 reads representing ~160× genome coverage. The genomes were assembled using a combination of *de novo* assembly with NGen version 4 (DNAStar, Madison, WI) and comparisons with available *P. agglomerans* genomes (11–13) using Mauve version 2.3.1 (14). The final assembly consists of a draft chromosome (16 contigs for a total 4,034,977 bp) and two closed plasmids, pPag1 (182,134 bp) and pPag3 (558,805 bp). All sequences were annotated automatically using GenDB (15), with manual optimization (16).

The plasmids pPag1 and pPag3 are relatives of the respective *Pantoea vagans* C9-1 plasmids (16). Plasmid pPag3 is a group I member of the large *Pantoea* plasmid 1 family (LPP-1) of plasmids, most closely related to the plasmids of other sequenced *P. agglomerans* strains (11). Plasmid pPag1 is present in all *P. agglomerans* genomes studied to date but also shows some variation between strains.

Comparative analysis using EDGAR (17) confirmed that the genomes of *P. agglomerans* strains are highly collinear. A distinct genomic island integrated in the *mutS* N-terminus was identified containing the pantocin A biosynthetic genes (18), one potential mechanism of action in biocontrol. Overall, this genomic island is related to the pantocin A genomic island of *P. vagans* C9-1, with slight variations (16).

The genome sequence of *P. agglomerans* P10c will give us the opportunity to improve strain-specific fingerprinting important for registration and intellectual property protection of active biological agents in commercial biocontrol products. Moreover, this provides a foundation to elucidate mechanisms of action and the regulation of antimicrobial compound biosynthesis, which in turn can be exploited to improve practical and commercial value through the optimization of formulation technology, performance reliability, and efficacy of this biocontrol strain.

### Nucleotide sequence accession numbers.

The whole-genome shotgun project of *P. agglomerans* P10c has been deposited at DDBJ/EMBL/GenBank under the accession no. LIME00000000. The version described in this paper is version LIME01000000. The finished plasmid sequences received accession numbers LIME01000017 (P10c pPag1) and LIME01000018 (P10c pPag3).
